# A High-Resolution Reflective Microwave Planar Sensor for Sensing of Vanadium Electrolyte

**DOI:** 10.3390/s21113759

**Published:** 2021-05-28

**Authors:** Nazli Kazemi, Kalvin Schofield, Petr Musilek

**Affiliations:** 1 Electrical and Computer Engineering, University of Alberta, Edmonton, AB T6G 1H9, Canada; nazli@ualberta.ca (N.K.); kschofie@ualberta.ca (K.S.); 2 Applied Cybernetics, University of Hradec Králové, 500 03 Hradec Králové, Czech Republic

**Keywords:** microwave sensor, negative resistance, vanadium redox flow batteries, reflective sensor

## Abstract

Microwave planar sensors employ conventional passive complementary split ring resonators (CSRR) as their sensitive region. In this work, a novel planar reflective sensor is introduced that deploys CSRRs as the front-end sensing element at $f_{\mathrm{res}}=6$ GHz with an extra loss-compensating negative resistance that restores the dissipated power in the sensor that is used in dielectric material characterization. It is shown that the $S_{11}$ notch of −15 dB can be improved down to −40 dB without loss of sensitivity. An application of this design is shown in discriminating different states of vanadium redox solutions with highly lossy conditions of fully charged $V^{5+}$ and fully discharged $V^{4+}$ electrolytes.

## 1. Introduction

During the past decade, microwave planar resonators have been found to be highly suitable for sensing applications, mainly due to their compact size, high sensitivity, low manufacturing cost, and high design flexibility [1,2,3,4,5,6,7,8,9,10]. Split ring resonators (SRR), along with their complementary version (CSRR), are the main building blocks of these sensors [11]. They provide regions that are highly sensitive to capacitive and resistive variations in the surrounding environment [12]. These metamaterial-inspired particles are used in liquid characterization [13,14,15,16,17,18,19,20,21,22,23], gas sensing [24], mechanical deformation sensing [25], temperature sensing [26,27], etc. However, they suffer from low-to-moderate quality factors that limit their applications to low-loss material sensing. Several loss-compensation techniques have been reported in the recent literature that demonstrate the impact of a negative resistance circuit on the sharpness of the transfer function of the sensor. Positive feedback is a method of recovering the lost electric field distribution, while it requires excessive components such as phase shifters in some cases [28]. Negative resistance has also shown its applications in sensing. However, possible oscillations should be considered a result of parasitic elements at high frequencies [29,30]. This work introduces a novel circuitry to cope with the excessive losses of the circuits with a negative resistance coupled to a CSRR. Therefore, the sensor, loaded with lossy medium, is compensated and its dynamic range increases with no adverse impact on the sensitivity. It is shown that the same resonance profile in terms of reflection parameter is achievable with a higher quality factor and deeper $S_{11}$ notch, and that this scheme is electronically controllable for various materials under test (MUT) with an arbitrary loss factor.

Vanadium redox flow batteries (VRFB) utilize the oxidation states of vanadium. Solid-state reactions are eliminated, and the VRFB electrodes are not prone to damage during deep discharge in contrast with intercalation-type materials used in other battery technologies [31]. The single-metal electrolyte system also reduces the cross-contamination issues that plague other types of redox flow batteries, and has superior stability and reversibility compared to other redox couples such as $C_{r}\left(\mathrm{III}\right)/C_{r}\left(\mathrm{II}\right)$ [31,32,33]. These factors dramatically improve the lifetime of VRFBs and reduce their maintenance requirements. Due to their low energy density, they are limited to stationary applications such as backup power supplies or telecommunication applications.

Capacity loss due to imbalanced material transfer through the membrane can be easily mitigated via periodic remixing of the anolyte and catholyte [34], or automatically via a hydraulic shunt [31].

Capacity loss due to side reactions (e.g., $V^{2+}$ oxidation) must be corrected via chemical or electrochemical rebalancing of the oxidation states [31,35,36]. Accounting for these losses allows the electrolyte to be used and its indefinite life to be maintained [36]. To achieve this, a suitable monitoring method is required to detect state of charge imbalances. Open-circuit voltage is one of the most widely used techniques; however, this produces inaccuracies due to the incomplete representation of the electrode potential, and is only accurate in a balanced system [35,36,37].

Conductivity probes have been successfully used to measure the state of charge (SOC) of both half-cells [31]. This is one of the main techniques, usually requiring a separate REDOX cell operating at zero current. When both half-cells are balanced, the terminal voltage can be correlated to SOC. However, side reactions such as precipitation or $V^{2+}$ oxidation may break the balance, and the total open-circuit potential does not reflect these imbalances [38]. This method has been used to estimate SOC, but requires calibration for varied electrolyte compositions. Errors may also be introduced due to temperature fluctuations during operation. A four-pole device—an improvement upon half-cells—has two inner poles to measure the potential drop across the electrolyte when a current is passed through the outer poles. Electrode polarization errors are eliminated, but calibration is needed for varied electrolyte compositions. It is also very sensitive to the applied current (poor voltage sensitivity when low, inaccurate measurements when high) and was created to reduce polarization errors [34]. Additionally, this method may produce measurement errors due to its temperature dependence. Similar errors exist when density measurements for SOC monitoring in lead-acid batteries are used [38], and at the same time requires extensive calibration measurements. Reference electrodes implemented in both half-cells allow imbalances to be detected. However, estimation errors arise due to impurities in the reference electrodes. Additionally, recalibration during battery operation is required due to electrode potential drift. Similar to conductivity, this method is composition- and temperature-dependent.

This work presents a novel approach for monitoring losses in the electrolyte solution using a high-resolution microwave sensor that enables a noncontact sensing scheme. In this method, the sensor is not in direct contact with the electrolyte, thus reducing the temperature and displacement dependency. Furthermore, this low-cost design provides a long-lasting sensing platform due to the low maintenance required as an immediate result of the contactless measurement. High-sensitivity CSRR resonators are employed to extract minute changes inside the lossy solutions.

## 2. Sensor Design and Analysis

**Figure 1:**
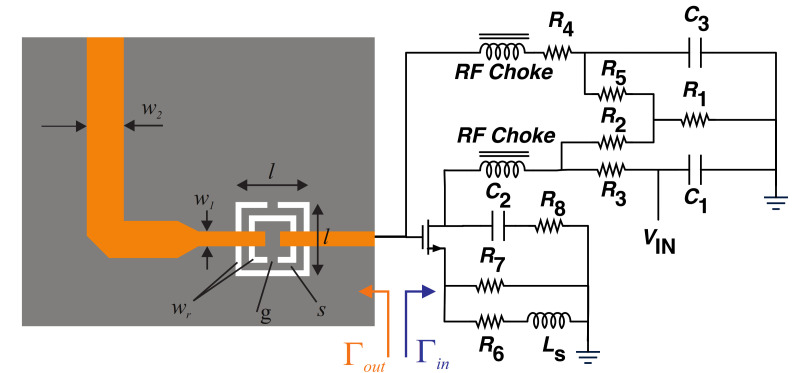
Schematic of the reflective sensor.

A CSRR was designed on Rogers 5880 substrates, with dielectric properties of $\varepsilon_{r}$ = 2.2, $tan\left(\delta \right)$ = 0.0009, and thickness of 0.8mm, which was the main sensing element of the reflective sensor. Its loss was modified using active circuitry on one end and it was interrogated from the other end. The choice of frequency for CSRR is tied to the dielectric properties of the loss of MUT that decreased with frequency. Thus, a relatively high value of 6 GHz was chosen, considering the fabrication tolerances of small size features. Moreover, to increase the sensitivity of the sensor, its loss was compensated with a negative resistance circuit using ATF34143 FET (Avago, San Jose, CA, USA), as shown in Figure 1. It should be noted that the sensor sensitivity will be intact as loss-compensation does not affect the frequency shifts of the sensor [2,39].

DC biasing of this PHEMT transistor uses feedback from drain to gate (through resistive division of $R_{1}/R_{2}$) with a resistive source ($R_{6}=R_{7}$) to enable negative $V_{gs}=-0.63$ V and ensuring $I_{d}=25$ mA, all with a single positive bias voltage of $V_{in}=6$ V (see Table 1 for full details). According to Barkhausen criterion, oscillations at the gate node of the circuit are triggered as long as the reflections from either side follow:

(1) $$|\Gamma_{in}\left|\times \right|\Gamma_{out}|>1.$$

**Figure 2:**
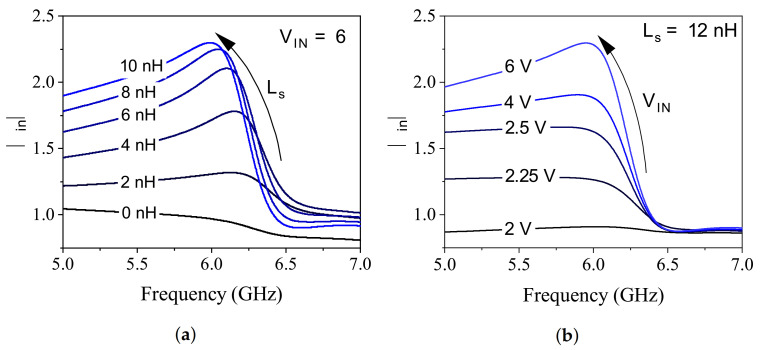
(**a**) Impact of inductive generation on $|\Gamma_{in}|$. (**b**) Bias voltage on $|\Gamma_{in}|$

However, partial compliance with this notion is enough for the circuit to compensate the losses occurred within CSRR using the negative resistance, wherein the circuit is not entering an oscillation mode. Loss compensation in the CSRR can be viewed as the level of restoring the lost power in the resonator at the frequency of resonance, that is directly modifying the depth of reflection. Therefore, the main goal of this study is to decrease the depth of $S_{11}$ notch, where the small capacitive/resistive variations brought about by the MUT are considerably distinguishable. To this end, the MOSFET’s stability was modified using a source degeneration inductor Ls, whose tuning resulted in increased reflection from the gate, also shown as $\left|\Gamma_{in}\right|$ in Figure 1. This is elaborated in Figure 2a, where the reflection at ∼6 GHz improves when increasing the inductive degeneration at the source that reaches 2.28 at $L_{s}∼10$ nH. Once the magnitude of reflection becomes larger, the multiplication in Equation (1) also improves towards unity considering that the reflection from CSRR is typically low in resonance. Once the circuit reflection improves from ∼1 with no $L_{s}$ up to 128% with $L_{s}=10$ nH, the negative resistance initiated by this node also increases. The bias voltage importance in this regard also provides another electronically tunable level of loss compensation. Figure 2b illustrates that the level of compensation can be controlled with a positive bias voltage, which enables applications related to different lossy environments, suitable for a wider range of materials.

**Figure 3:**
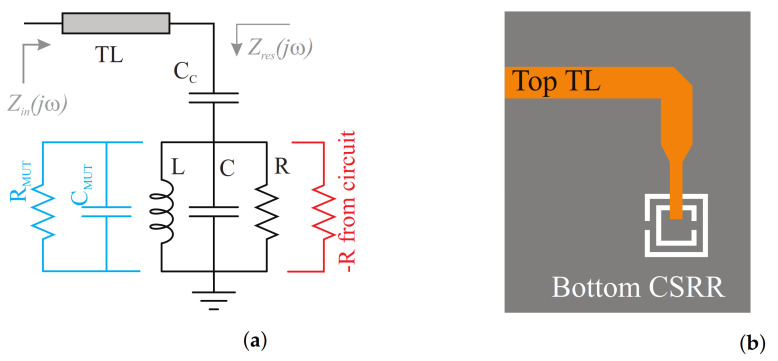
(**a**) Circuit model with extracted parameters as *L* = 0.59 nH, *C* = 1.037 pF, *C_C_* = 0.18 pF, and *R* = 1.47 kΩ. (**b**) CSRR interrogation.

The electrical circuit model of the CSRR is elaborated in this section. Since the electrical size of the CSRR is small with respect to the guided wavelength, the structure can be described with equivalent lumped elements. In this model, the input transmission line from the measurement port is considered, which is loaded with the CSRR as half of the circuit. The other half is purely intended to provide negative resistance to compensate for the lost power, as shown in Figure 3a,b.

In this model, the resonant tank is represented by parallel *R* as resistance, *L* as inductance, and *C* as capacitance. This resonator was coupled capacitively to the single transmission line through the substrate, shown as capacitor $C_{C}$. The resonator can potentially be loaded with an external MUT that is shown with capacitance $C_{\mathrm{MUT}}$ and a lossy component $R_{\mathrm{MUT}}$. To extract the model parameters, full-wave simulations in HFSS are required. In a simpler view of the model, $Z_{in}$ depends highly on $Z_{\mathrm{res}}$ as the transmission line only shifts the phase of impedances according to its electrical length. Therefore, S-parameter measurement and analysis were conducted by de-embedding the transmission line length. Since there are four elements in the representation of $Z_{\mathrm{res}}$, four equations are required to accurately determine the constituent parameters. Ignoring *R* for simplicity, $Z_{\mathrm{res}}$ can be reduced to

(2) $$Z_{\mathrm{res}}=j\frac{L\left(C+C_{C}\right)\omega^{2}-1}{C_{C}\omega (1-LC\omega^{2})\prime }$$

which depicts two characteristic frequencies—$f_{z}$, the frequency that nulls $Z_{\mathrm{res}}$, and $f_{o}$, the frequency that nulls $Y_{\mathrm{res}}=1/Z_{\mathrm{res}}$—as follows:

(3) $$f_{0}=\frac{1}{2\pi \sqrt{LC}\prime },$$

(4) $$f_{z}=\frac{1}{2\pi \sqrt{L(C+C_{C})}}.$$

**Figure 4:**
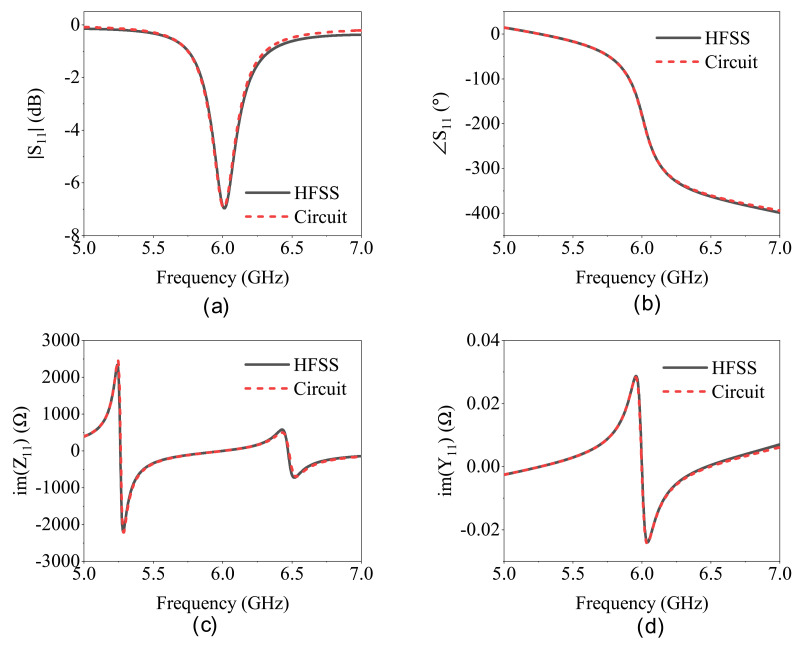
Comparison between full-wave simulations of CSRR and its circuit model in (**a**) $|S_{11}|$, (**b**) $∡S_{11}$, (**c**) im $\left(Z_{11}\right)$, and (**d**) im $\left(Y_{11}\right)$.

These values can be verified from full-wave simulation results. At $f_{z}$, the transmission line terminates at an open end due to the resonance, which coincides with the dip frequency of $S_{11}$. Now, considering the impact of parallel resistance *R* on the depth of the reflection coefficient (see Figure 4a), it can be inferred from the equivalent impedance seen from $Z_{\mathrm{res}}$ node at resonance frequency $f_{z}$, where the imaginary part of $Z_{\mathrm{res}}$ becomes zero and only the real part remains as:

(5) $$S_{11}\left(f_{z}\right)=\frac{RC_{C}/\left(C+C_{C}\right)-Z_{0}}{RC_{C}/\left(C+C_{C}\right)+Z_{0}}.$$

This resistive impedance, seen from $Z_{\mathrm{res}}$ node, impacts the depth of the resonance seen at $f_{z}$. It becomes an ideally reflection-less resonance when the impedance $Z_{\mathrm{res}}=Z_{0}$ as the transmission line impedance. The last expression is obtained from the $S_{11}$ phase response at the frequency of resonance $f_{o}$, wherein only *R* remains in series with $C_{C}$ with parallel LC resonance when viewed from the $Z_{\mathrm{res}}$ node. This phase can be calculated as:

(6) $$∡S_{11}\left(f_{0}\right)=tan^{-1}\left(\frac{2RC_{C}\omega }{1+R^{2}-Z_{0}^{2}\omega^{2}C_{C}^{2}}\right).$$

The extracted parameters are given in the caption of Figure 3. Figure 4 represents the reflection coefficient $S_{11}$ in the magnitude (Figure 4a), phase (Figure 4b), imaginary part of impedance (Figure 4c), and admittance (Figure 4d) of the circuit model in the Advance Design System (ADS). At frequencies that cover the resonance, these coefficients are greatly congruent with the full-wave simulation in HFSS.

**Figure 5:**
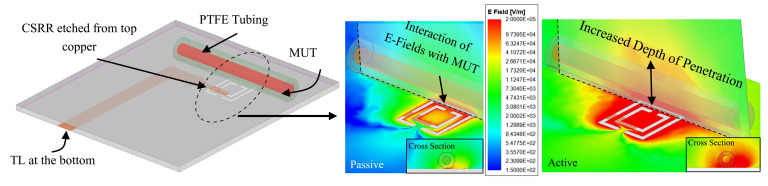
Simulation setup in HFSS with ∣E∣ field demonstration on the surface of the CSRR as well as the cross-section of MUTs above it.

The simulation setup in HFSS consists of the etched ground, with dimensions listed in Table 1, loaded with a PTFE tubing that is flush with the surface of the sensor, as shown in Figure 5. The curved cylindrical shape of the tube limits its maximum interaction with the planar surface of the sensor. However, the loss-compensation scheme reinforces the electromagnetic fields around the CSRR that propagate into the tube with MUT inside [40,41,42]. The correct orientation of the tube with respect to the slots of CSRR has been demonstrated in previous works [43] to be parallel with the longest slot of the outer ring. This ensures the maximum electric field generated at the center of the slot to interact with the MUT. The magnitude of the electric field interaction with MUT is illustrated in Figure 5, which also shows that the highest fields (with red color) are present at the location of the tube. This configuration reveals the highest sensitivity, where the tube covers the longest slot. The plotting plane crossing the tube and MUT depicts |E|-fields emanating into the tube along the long slot.

**Figure 6:**
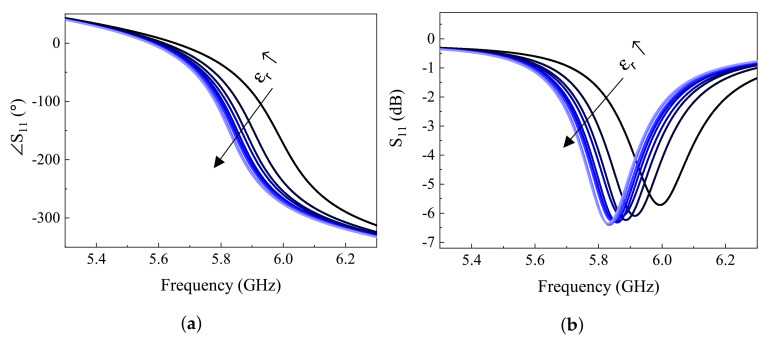
(**a**) Phase and (**b**) magnitude of S11 response in simulation of various MUT properties, assuming tan(*δ*) = 0, while $\varepsilon_{r-\mathrm{MUT}}$ = 10:10:80 and $\varepsilon_{r-\mathrm{MUT}}$ = 1.

Performance of the proposed sensor was evaluated using simulations of MUT with various dielectric properties inside the tube. In the first case, the losses in MUT were ignored and only permittivity was varied from 10 up to 80 with increments of 10 as well as $\varepsilon_{r}=1$, as shown in Figure 6. It is clear that the resonance frequency, also known as $f_{z}$ in this analysis, reduces due to the added CMUT, as shown in Figure 3a. This behavior can also be expressed in terms of the resonance frequency change, assuming a generic planar sensor with substrate permittivity $\varepsilon_{r-\mathrm{sub}}=1$, whose whole slots are covered by MUT with $\varepsilon_{r-\mathrm{MUT}}=1$. Then, $f_{z}$ can be shown as [5]:

(7) $$f_{z}=\frac{1}{2\pi \sqrt{L\left(C_{C}+C\frac{\varepsilon_{r-\mathrm{sub}}+\varepsilon_{r-\mathrm{MUT}}}{\varepsilon_{r-\mathrm{sub}}+1}\right)}}.$$

This shows an inverse relationship between MUT permittivity and the resultant resonance frequency. For more clarity, it can be expressed in terms of sensitivity *S* as follows:

(8) $$S=\frac{\partial f}{f_{z_{0}}\partial \varepsilon_{r-\mathrm{MUT}}}=-\frac{1}{2}\frac{C}{C_{C}\left(\varepsilon_{r-\mathrm{sub}}+1\right)+C\left(\varepsilon_{r-\mathrm{sub}}+\varepsilon_{r-\mathrm{MUT}}\right)},$$

where $f_{z_{0}}$ represents the initial resonance frequency of $\epsilon_{\mathrm{MUT}}$ = 1. For small coupling between the feeding transmission line and the CSRR, where $C_{C}\ll C$, this can be simplified as:

(9) $$S=-\frac{1}{2}\frac{1}{\left(\varepsilon_{r-\mathrm{sub}}+\varepsilon_{r-\mathrm{MUT}}\right)}.$$

**Figure 7:**
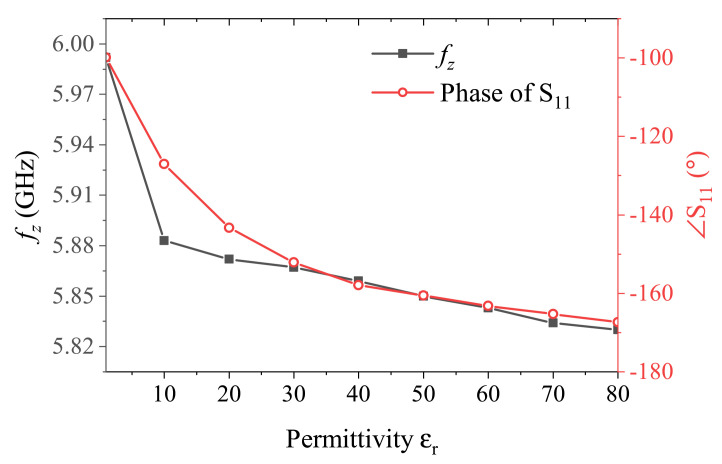
Comparison between frequency and phase sensitivity.

This equation shows that the sensitivity has an inverse relationship with the additional MUT permittivity and that it flattens out at values $\varepsilon_{r-\mathrm{MUT}}\approx 10\;\varepsilon_{r-\mathrm{sub}}$. This is the internal characteristic of planar CSRR, whose sensitivity is not as good at high permittivity variations. Therefore, another attempt was made to exploit the phase feature of the sensor. A comparative view of the resonance frequency of $S_{11}$, also known as $f_{z}$, with phase of $S_{11}$ extracted at $f_{z}$ corresponding to various MUTs is shown in Figure 7. It is obvious that the extraction of a closed form for phase is cumbersome compared to the resonance frequency. It showcases that the linearity of the frequency of resonance drops much quicker than that of the phase measured at the corresponding frequencies, as $f_{z}$ starts a shallow slope as soon as the MUT permittivity crosses ∼10, while phase holds higher linearity—though with slightly less sensitivity—up to a MUT permittivity value of ∼30. This improvement in the linearity helps achieve higher effective sensitivity at a broader dynamic range of MUT. Therefore, the purpose of this work is to delve into analyzing the phase response to resolve small deviations in MUT properties. Corresponding phase behavior of this simulation is also given in Figure 7.

**Figure 8:**
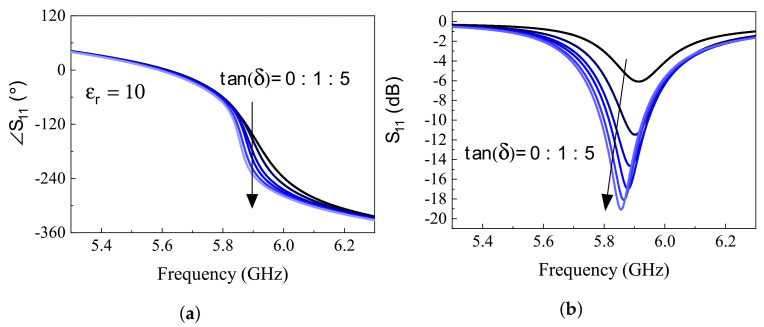
(**a**) Phase and (**b**) magnitude of *S*_11_ response in simulations of various MUT properties, assuming $\varepsilon_{r-\mathrm{MUT}}$ = 10, while tan(*δ*) = 0:1:5.

**Figure 9:**
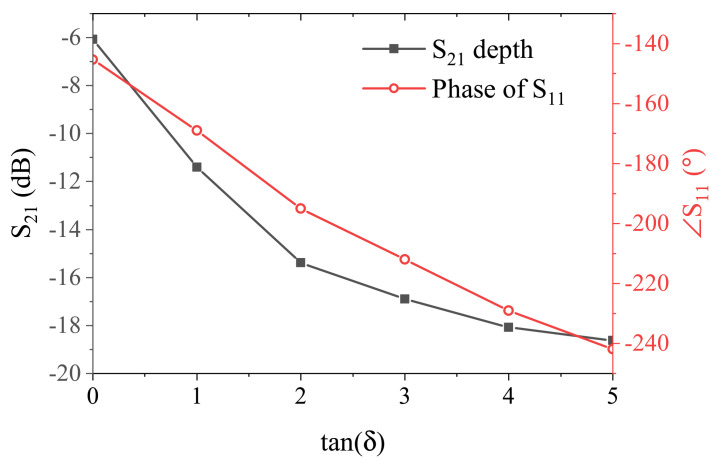
Comparison between $S_{11}$ depth and phase sensitivity.

Another exploration of the sensor response is on its behavior with respect to the loss of MUT. Figure 8 depicts the results for both magnitude and phase of S11 with MUT permittivity being constant as $\varepsilon_{\mathrm{MUT}}=10$, while the loss tangent tan(δ) varies from 0 to 5 with increments of 1. It is noteworthy that loss in the MUT acts as a resistance $R_{\mathrm{MUT}}$ parallel to the internal resistance of the sensor R, which mostly $R_{\mathrm{MUT}}<R\ll Z_{0}$ due to the lossy nature of MUT. This results in a smaller equivalent resistance, which is closer to the line impedance $Z_{0}$ when viewed from $Z_{\mathrm{res}}$ node, thus, better matching is achieved with deeper $S_{11}$. This notion is captured in Figure 8, where lossy MUT is shown to increase the slope of phase response at the resonance frequency and the $S_{11}$ notch become deeper. Figure 9 depicts the performance comparison between the depth of $S_{11}$ and the phase of the reflection at initial resonance frequency of the passive mode. It turns out to follow the trend as discussed for permittivity measurements, where the phase response exhibits more linearity compared to the depth of $S_{11}$ with tan(δ), ranging from 0 to the fairly high value of 5 vs. the nonlinear saturation mode occurring for $S_{11}$ depth starting around tan(δ) = 2.

## 3. Vanadium Electrolyte (MUT)

A VRFB utilizes two soluble redox couples, allowing a fully liquid-state system to be created based on a single metal. These active materials are typically dissolved in an acidic electrolyte such as sulfuric acid, with a stabilization agent such as HCl or H_3_PO_4_ used to improve the vanadium ion’s solubility (and consequently the electrolyte’s energy density). The electrolyte was pumped from two reservoirs through a 2.5 kW, 40-cell stack at a nominal flow-rate of 3 L/min. A 5000 VA Quattro inverter (Victron Energy, Almere, Netherlands) was used to apply current during the charging process. A nominal current of 30 A was applied until a stack voltage of 62 V was reached. The stack was then charged under constant-voltage mode until the current reduced below 1 A. At the end of this process, V^2^+ and V^5^+ are present in the negative and positive reservoirs, respectively. Discharging was performed through a series of resistors (1.8 ohm constant), resulting in a maximum current of 45 A. Discharging was stopped after reaching a cutoff voltage of 30 V. Battery monitoring was performed using a multifunction I/O device USB-6211 (National Instruments, Austin, TX, USA). At the end of this discharging process, V^2^+ and V^3^+ are present in the negative and positive reservoirs, respectively.

During charging, the catholyte is converted from V^4^+ to V^5^+ Equation (2), while the anolyte is converted from V^3^+ to V^2^+ Equation (3) [5]:

(10) $${VO}_{\left(aq\right)}^{2+}+H_{2}O_{\left(l\right)}\to {VO}_{2(aq)}^{+}+2H_{\left(aq\right)}^{+}+e^{-},$$

(11) $${VO}_{\left(aq\right)}^{3+}+e^{-}\to {VO}_{\left(aq\right)}^{2+}.$$

In this work, the catholyte solution was studied in two extreme cases (fully-charged and -discharged) with microwave planar sensors to enable a noncontact sensing scheme.

## 4. Measurement Results and Discussion

**Figure 10:**
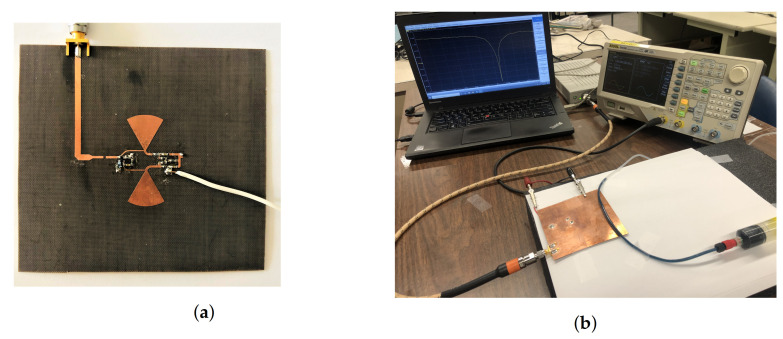
(**a**) Fabricated sensor, top view; (**b**) test setup.

The proposed sensor was fabricated on Rogers 5880 substrates with dielectric properties of $\varepsilon_{r}=2.2$, tan(δ)=0.0009, and thickness of 0.8 mm (see Figure 10). All transmission lines as well as the circuit elements are on the top side of the substrate, while the CSRR is etched out of the ground plane on the other side. Radial stubs terminating at high-impedance transmission lines comprise RF chokes. CSRR was fed with narrow transmission lines to reduce the coupling and achieve a high-quality-factor resonance.

**Figure 11:**
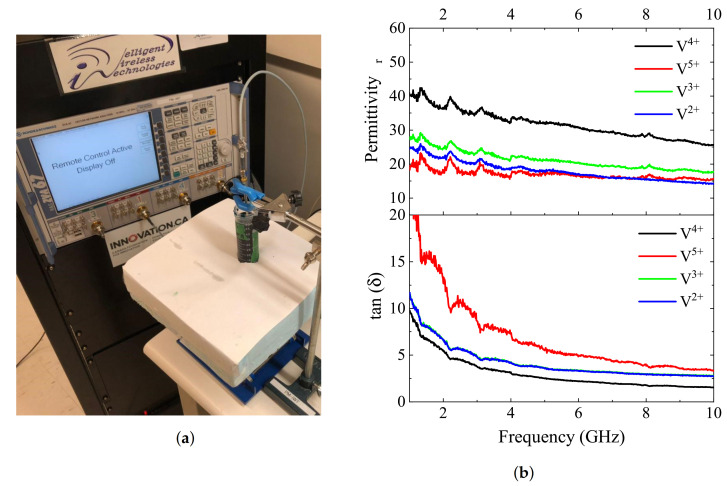
(**a**) Dielectric probe test setup, (**b**) Dielectric probe measurement results for permittivity and loss tangent of vanadium electrolytes.

Dielectric properties of vanadium electrolytes were measured using the standard dielectric probe setup shown in Figure 11a,b for states of both charged and discharged solutions V^2+^, ^3+^, ^4+^, ^5+^ within a frequency range of 1–10 GHz. Loss tangent information reveals the level of loss in these solutions merely due to the high concentrations of ions, and it is clear that the permittivity as well as the loss tangent values differ between V^4+^ and V^5+^ more significantly than those of V^2+^ and V^3+^. This broadness in the permittivity range implies better sensitivity when monitoring the solution containing V^4+^/V^5+^ with a microwave sensor.

**Figure 12:**
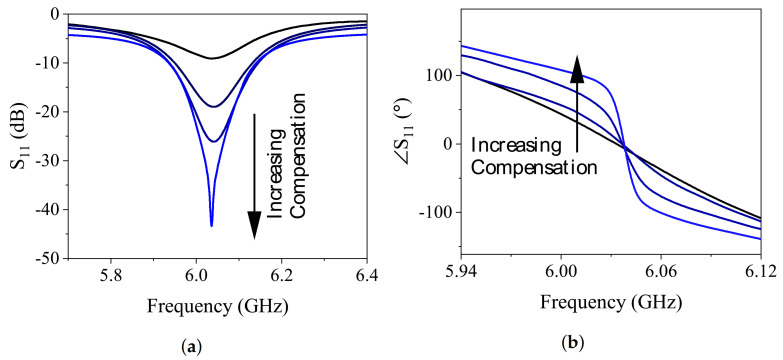
Reflection parameter modified due to negative resistance in (**a**) magnitude and (**b**) phase.

The sensor’s reflection parameter was studied by applying voltage to the input with no MUT present, and the depth of $S_{11}$ increased at higher voltages, as shown in Figure 12a. In addition, the slope of the phase response, shown in Figure 12b, becomes steeper along loss-compensation, translating to an improved quality factor. This also proves the impact of negative resistance on the sensor’s energy restoration. The impact of loss compensation on the resolution can be verified by examining the quality factor of the sensor. The sharper the resonance profile, the lower the concentration change that can be inferred since the reflection profiles are more distinct. The 3-dB bandwidth of the sensor, as well as the depth of $S_{11}$, were computed at different conditions of passive and various loss-compensation modes (i.e., denoted by $V_{in}$ = 2, 4, and 6 V), as shown in Table 2. The 3-dB bandwidth was calculated with respect to the frequency span at 3-dB above the notch depth, and it is useful to assess the sharpness of the reflection curves. The lower the bandwidth, the more distinct the profile becomes, helping to sense small changes. It is evident that the higher compensation level reduces both the reflection depth and its 3-dB bandwidth almost 48 times compared with the passive condition. To assess the sensor’s performance, the following sections provide measurement results with respect to common liquids as well as the vanadium redox solution.

### 4.1. Common Liquid Measurement

To evaluate the sensor’s response, common liquids including IPA, ethanol, methanol, and water were prepared. The sensor was measured using S5065 VNA (Copper Mountain). The MUT was passed through a PTFE tubing that was secured to the sensor to ensure repeatability and reliability, as shown in Figure 10b. All liquids were injected individually, and the tube was purged with water after each measurement to remove leftover chemicals.

**Figure 13:**
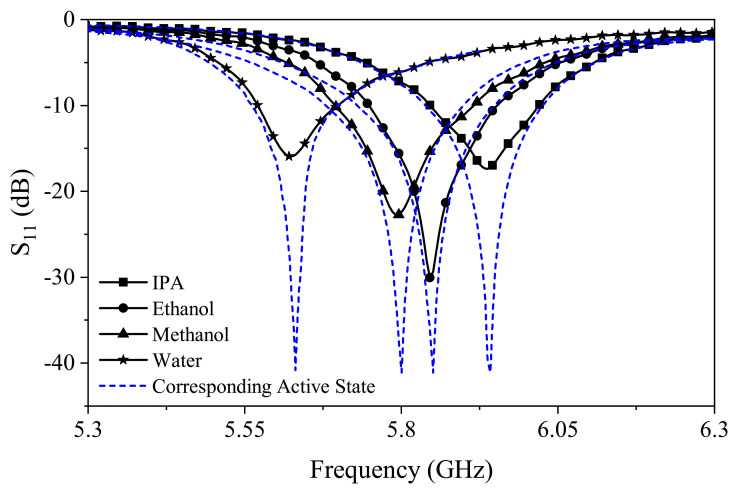
Common liquid measurement with/without loss compensation.

Figure 13 presents the reflection from the sensor under two conditions: when the negative resistance is not triggered (solid black line), and when the negative resistance is employed to compensate the loss (dashed blue line). In each MUT test, a specific bias voltage (refer to Figure 2b) was applied to deepen the $S_{11}$ profile, depending on the dielectric property of each sample. The results show that the proposed design, when filled with an MUT, is not only able to restore the original $S_{11}$ depth before filling, but also to improve the depth arbitrarily. It is clear that the sensor shows high performance when retrieving the resonance condition for lossy environments (e.g., IPA and water) from $S_{11}∼15$ dB down to ∼40 dB. Having said that, the frequency of resonance at each measurement was not impacted by the negative resistance, which suggests no adverse loading impact of the circuit on the resonator. This way, one can utilize the sensor at various conditions with substantially increased resolution to recognize minute frequency/amplitude variations.

### 4.2. Measuring Vanadium Redox Solutions

**Figure 14:**
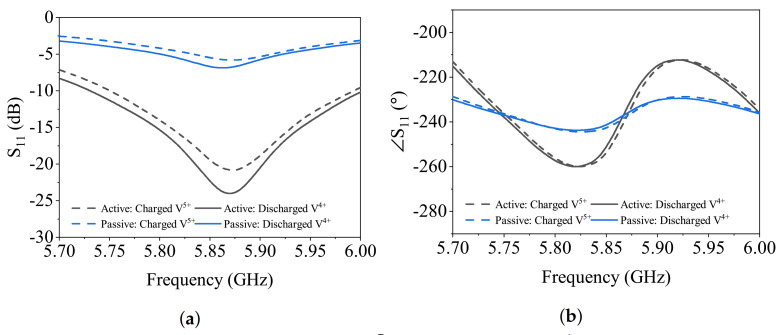
Measurement result for charged (*V*^5+^) and discharged (*V*^4+^) redox solutions: (**a**) mmagnitude and (**b**) phase.

Once the sensor was evaluated with common chemicals, the vanadium redox liquids were exposed to the sensor with the same tubing configuration and injection procedure. Two extreme cases of vanadium redox liquids include positively charged and discharged solutions. Based on the dielectric properties of these solutions measured with a dielectric probe at 6 GHz, the charged solution $V^{5+}$ is characterized with $\varepsilon_{r}=17-j85$, while the discharged solution $V^{4+}$ is measured $\varepsilon_{r}=30-j66$. Although their permittivity values are considerably different, the magnitude of loss in either state of Vanadium redox process is high enough to overshadow the permittivity that prevents the regular mode of sensor operation from distinguishing them. Therefore, negative resistance was activated to enhance the sensor dynamic range. Figure 14 showcases the difference in $S_{11}$ profile for the two liquids, which is in agreement with their dielectric permittivity. The charged solution lowers the resonance frequency more because of its higher permittivity (30 vs. 17 of discharged).In both cases of amplitude and phase of reflection, the active sensor reveals more distinct graphs, even though the sensitivity is intact.

## 5. Conclusions

In this work, a complementary split ring resonator is used as the planar microwave sensing element for material characterization. However, its application in a lossy environment of vanadium electrolyte solutions urged feeding this element with a negative resistance circuit to restore the lost power and compensate the loading impact from the lossy MUT. For this reason, the CSRR at 6 GHz is accompanied with a source-degenerated PHEMT. Its performance has been verified using various common chemicals including IPA, ethanol, methanol, and water. The sensor has shown considerable discrimination power for the highly lossy ion-based vanadium redox solution between the two states of fully discharged and fully charged. This sensor property is promising for its application in monitoring the state of charge in the harsh environment of vanadium electrolyte. The next step toward the operational deployment of the sensor will be a detailed examination of its discrimination ability for intermediate levels of VRFB charge.

## Figures and Tables

**Table 1:** Dimensions of the sensor with component values.

$R_{1}$	$R_{2}$	$R_{3}$	$R_{4}$	$R_{5}$	$R_{6}=R_{7}$
220 kΩ	1.8 kΩ	50 Ω	50 Ω	10 Ω	75 Ω
**$C_{1}=C_{3}$**	**$C_{2}$**	***l***	**$w_{1}$**	**$w_{2}$**	**s=g**
0.1 μF	100 μF	5 mm	1 mm	2 mm	0.4 mm

**Table 2:** Performance comparison of passive and active sensor values.

Parameter	Passive	$V_{\mathrm{in}}=2V$	$V_{\mathrm{in}}=4V$	$V_{\mathrm{in}}=6V$
Depth of $S_{11}$	−9	−18.9	−26	−43.3
3-dB BW	175	83	57.5	3.6

## Data Availability

The data are not publicly available due to data privacy restrictions.
